# Rapid Enzymatic Response to Compensate UV Radiation in Copepods

**DOI:** 10.1371/journal.pone.0032046

**Published:** 2012-02-22

**Authors:** María Sol Souza, Lars-Anders Hansson, Samuel Hylander, Beatriz Modenutti, Esteban Balseiro

**Affiliations:** 1 Laboratorio de Limnología, INIBIOMA (CONICET-UNComahue), Bariloche, Argentina; 2 Institute of Biology/Aquatic Ecology, Ecology Building, Lund University, Lund, Sweden; Institute of Marine Research, Norway

## Abstract

Ultraviolet radiation (UVR) causes physical damage to DNA, carboxylation of proteins and peroxidation of lipids in copepod crustaceans, ubiquitous and abundant secondary producers in most aquatic ecosystems. Copepod adaptations for long duration exposures include changes in behaviour, changes in pigmentation and ultimately changes in morphology. Adaptations to short-term exposures are little studied. Here we show that short-duration exposure to UVR causes the freshwater calanoid copepod, *Eudiaptomus gracilis*, to rapidly activate production of enzymes that prevent widespread collateral peroxidation (glutathione S-transferase, GST), that regulate apoptosis cell death (Caspase-3, Casp-3), and that facilitate neurotransmissions (cholinesterase-ChE). None of these enzyme systems is alone sufficient, but they act in concert to reduce the stress level of the organism. The interplay among enzymatic responses provides useful information on how organisms respond to environmental stressors acting on short time scales.

## Introduction

Over the course of normal life, organisms face multiple threats, and these vary on different time scales. One way to deal with this variety of fluctuations is to have traits that are induced only when needed. UVR requires different types of responses because it acts both on long (i.e. seasonal changes) and on short-term scales (e.g. cloud fluctuations). The wavelengths of UVR that currently reach the Earth's surface, independent of any ozone depletion events, cause severe cellular damage [1], most notably, DNA damage. The link between sunlight and potential damage is well established for a variety of aquatic organisms [1], [2], [3]. Early studies have indicated that the mechanisms of damage and the processes of repair at the cellular level are similar for prokaryotes and eukaryotes [4]. Zooplankton must integrate environmental information and use a variety of mitigating strategies to counteract the damaging effects of UVR. Such responses include photoprotective compounds (mycosporine-like amino acids and pigments) [5], behavioural responses [6], and photoenzymatic repair (PER; [7]). At the organism level, a response depends on and must be integrated with cell-level signals and conditions, such as oxidative stress damage, antioxidant defence concentrations, and enzymatic expression that are likely to be governed by a succession of changes at the molecular level acting at the time scale of hours. This hierarchical integration can extend further ecological fitness indicators, such as reproduction, survival, and responses to predation risk. For example, cell division, apoptosis, and growth factors must be precisely coordinated in order to guarantee the organism's success, which demonstrates the need for balance and harmonization between such conditions and effects [8].

Zooplankton may conform to long-term (seasonal) shifts in UVR intensity by adjusting their levels of photoprotective compounds, accomplishing this in days to weeks [9], [10]. Shorter-term shifts such as daily cycling of UVR can be handled with behaviour like vertical migration [9], [10], [11]. Further, in order to meet and counter the detrimental effects of rapid and strong fluctuations in UVR within a day, organisms may also take advantage of enzymatic responses at the cellular level. In a pioneering work, Borgeraas and Hessen [12] reported on diel variations in activity of the antioxidant enzymes in arctic *Daphnia*. Glutathione *S*-transferase (GST) is a detoxifying enzyme involved in the removal of reactive organic hydroperoxides. For zooplankton it represents a key enzymatic defence against reactive oxygen species (ROS) that are produced by UVR [13], [14]. In addition to its role in causing oxidative stress, UVR was recently characterized as one of the most important pro-apoptotic stimuli for crustaceans [15]. Apoptosis may play, in pluricellular organisms, an important role during developmental stages as embryogenesis and metamorphosis [16]. More specifically, during development many cells are produced in excess and eventually undergo programmed cell death, but in the interim they contribute to the ‘sculpturing’ of organs and tissues [17]. However, apoptosis will also play a protective role when eliminating damaged and unrecovered cells [18]. Among zooplankton, copepods are characterized by a complex development with different stages including nauplii and juveniles. Alteration in apoptosis processes during development could affect not only the individual and the population, but also the community, since these stages are crucial to community structure and energy transfer through the food web. Important in the process of apoptosis, caspases are a family of proteases that mediate cell death. In particular, caspase-3 (Casp-3) plays a central role in the apoptotic signalling network [19] and leads to DNA fragmentation and the cell's demise. Casp-3 and GST may represent early physiological responses for the mitigation of the detrimental effects of UVR and that could complement other UVR defences (photoprotective compounds) acting at longer time scales.

In addition to DNA damage, UVR can cause carboxylation of proteins and peroxidation of lipids [10], [20], and detrimental effects on physiological endpoints as respiration rates [21] and neurological dysfunctions like alterations in cholinesterase (ChE) activity [22], [23], among others. Hence, we include ChE activity as sensor of cellular damage in relation with the balance of protective systems of GST and Casp-3.

It is well known how organisms handle UVR threat that fluctuates over the long term, for example by accumulating photoprotective compounds [24], but the knowledge of responses at the cellular level to short term UVR threats is negligible. A rapid enzymatic response to handle fluctuations in UVR may be extremely important for the performance of the organism [14]. But all protective responses that involve phenotypic plasticity, such as in UVR protection, must undergo an initial time lag before they become effective [25], however this time-lag will vary greatly depending on the mechanism involved. Thus, we hypothesize that enzymatic responses to UVR stress will act on short time scales (hours), and that these responses vary according to the developmental stage of the organism. In order to test these hypotheses, a laboratory study was designed to assess the balance among different enzymes linked to important protective mechanisms, namely GST (defence) and Casp-3 (apoptosis), as fast and coordinated responses to UVR in two different developmental stages of the calanoid copepod *Eudiaptomus gracilis*. We provide the first evidence on how UVR affects Casp-3, as well as the interplay with other enzyme protective systems as GST.

## Materials and Methods

### Experimental design

For laboratory experiments, the calanoid copepod species *Eudiaptomus gracilis* was collected with a plankton net (125 µm mesh) from surface waters (0–0.5 m depth) of the small lake Dalby quarry, which is situated close to Lund in southern Sweden (55.67°N, 13.5°E). The quarry is about 10 m deep and has an absorption coefficient at 320 nm of 2.0 and a 1% attenuation depth of 1.56 m at that wavelength. Copepods were transported to the laboratory in containers with natural lake water at 18°C. Prior to the experiment, they were rinsed in filtered (10 µm) lake water.

We analysed the short-term changes in enzyme activities (GST, Casp-3 and ChE) at different UVR doses. 120 copepods were transferred to each of 48 plastic containers (200 mL) with filtered (80 µm) lake water. Six of these containers were immediately frozen (−80°C) as “time zero” (0 h). Another six replicates were wrapped with aluminium foil as Dark Control (DC). The other 36 containers were exposed to UVR for different periods (1, 2, 3, 4, 5, and 6 hours) at 18°C. This exposure resulted in cumulative doses of 38.9 kJ m^−2^, 77.8 kJ m^−2^, 116 kJ m^−2^, 155 kJ m^−2^, 194 kJ m^−2^, and 233 kJ m^−2^ of UVA, respectively. All dose measurements were made with the UV sensor (SUL 033; International Light, Newburyport, Massachusetts, USA). At the end of each incubation period, animals isolated from six containers (replicates) were separated and frozen (−80°C) until biochemical determination. DC containers were run under the same conditions throughout the 6 h of the experiment.

As UVR source we used eight UVA-340 fluorescent tubes (Q-Panel Lab Products, Cleveland, OH, U.S.A.) placed horizontally at 0.7 m from the containers. The UV spectrum of these light tubes closely resembles the solar spectrum between 290 and 350 nm (for full spectral analysis see [26]. However, by adding only PAR light a part of the long UVA (380–400 nm) was underrepresented in the exposure experiment. So the process of photorepair (PER) was probably underestimated.

Carotenoid extractions followed standard methods [6], [26], [27]. Before the sampling, animals were kept in tap water for at least 1 hour for gut evacuation and then measured at 40× magnification (Olympus SZ 40) before freezing at −80°C. Carotenoid samples were extracted in ethanol (95%) and quantified with a Lightwave II spectrophotometer (Biochrom, Cambridge) at 474 nm, the absorption peak for common copepod carotenoids, i.e. astaxanthin and its esters [28], [29], [30]. Measurements were done from top of head to the end of the furca. Carotenoid quantities were normalized to dry weight, which was calculated from published relationships between length and dry weight for the same copepod species [31].

### Enzyme activity determinations

In order to determine the enzymatic activity, the frozen specimens of *E. gracilis* from each treatment were rinsed twice with milliQ water, and separated on ice into two groups: juveniles and adults without eggs. Adults with eggs were scarce and hence excluded from the analysis. For GST and ChE activities, animals were homogenized using a glass-Teflon homogenizer with ice-cold 50 mM potassium phosphate buffer (pH = 7.7) containing 1 mM EDTA and 0.1% Triton X-100, according [32]. Homogenates were centrifuged at 10,000× g and 4°C for 10 minutes and supernatants used as enzyme sources.

Total cholinesterase (ChE) activity was determined at 25°C following the classic colorimetric method of Ellman et al. [33], at saturating concentrations of acetylthiocholine iodide as substrate and dithiobisnitrobenzoate (DTNB) as reagent and using absorbance at 240 nm. ChE activity was expressed as micromoles of product developed per minute per g of protein (µmol prod min−1 [g prot]). A linear increase in enzyme activity with increasing concentrations of samples proteins was verified (r^2^ = 0.962).

Total glutathione *S*-transferase (GST) activity was determined following Habig et al. [34]. In 100 mM phosphate buffer (pH = 6.5), with 1 mM of 1-chloro-2,4-dinitrobenzene (CDNB) in acetonitrile (1% v/v) and 1.2 mM glutathione GSH as substrates, activity was recorded using absorbance at 340 nm. Specific activity of GST was expressed in nanomoles of product developed per minute per mg of protein (nmol prod min^−1^ [mg prot]^−1^) at saturating substrate concentrations. A linear increase in enzyme activity with increasing concentrations of samples proteins was verified (r^2^ = 0.975).

The caspase-3 (Casp-3) activity was determined using, as substrate, the specific peptide Ac-DEVD- *p* nitroanilide (Ac-DEVD- pNA; Sigma kit: CASP 3-C). The fact that the core components of the cell death machinery are conserved through evolution [35], allowed us to determine Casp-3 activity in different stages of copepods with this kit. Briefly, animals were homogenized with lysis buffer containing 250 mM HEPES (pH = 7.4), 25 mM 3-[(3-Cholamidopropyl) dimethylammonio]-1-propanesulfonate (CHAPS), 25 mM dithiotreitol (DTT), and 2 µM leupeptin as protease inhibitor. Then the samples were held on ice for 20 minutes. Supernatants of homogenates (20000× g and 4°C for 15 minutes), were used as enzyme sources. Extracts were added to Caspase-3 reaction buffer (200 mM HEPES, pH 7.4, 1% CHAPS, 50 mM DTT, 20 mM EDTA) with Ac-DEVD- pNA substrate and then incubated at 37°C for 2 h. Unbound pNA concentration was determined as absorbance at 405 nm. The concentration of the pNA released from the substrate was calculated from the absorbance values at 405 nm based on a calibration curve prepared with pNA standard solutions (r^2^ = 0.9997). The specific activity of Casp-3 was expressed in µmol of product developed per minute per mg of protein (µmol prod min^−1^ [mg prot]^−1^) at saturating substrate concentrations.

Measurements of enzymatic activities were carried out using a Beckman Coulter DU800 spectrophotometer.

### Protein determination

The enzymatic activities were normalized to protein concentration. Protein determination was performed according to Lowry and co-workers [36] with bovine serum albumin as standard. The protein quantity per assay was 7.32±0.08 µg proteins per reaction for GST and ChE activities, and was 45.68±6.027 µg protein per reaction for Casp-3 determination.

### Data analysis

Results are expressed as mean ± standard error. Statistical significance was determined using a *t*-test and two way ANOVA (with copepod's stage and UVR doses as factors). When significant differences were obtained in the ANOVA, we applied an *a posteriori* multiple comparison Tukey test. All data fulfilled homoscedasticity and normality requirements of the statistical analyses. Quadratic and Linear regression were performed to characterize enzymatic patterns activity under UVR increasing doses and Pearson's Correlation was included to evaluate the relationship between GST and Casp-3 activity.

## Results

We observed that adults tended to have higher pigmentation per body mass unit than juveniles, but the difference was not statistically significant (*t* test *p* = 0.178). The average carotenoid pigment concentrations were 3.35±0.48 µg mg^−1^ dry mass in juveniles and 4.04±0.45 µg mg^−1^ dry mass in adults. In addition to slight differences in pigmentation, we ascertained distinctive effects on enzyme activities in two copepod stages as a result of the short-term exposure to increasing (cumulative) UVR doses. Each of the studied enzymes showed a different trend pattern.

**Figure 1:**
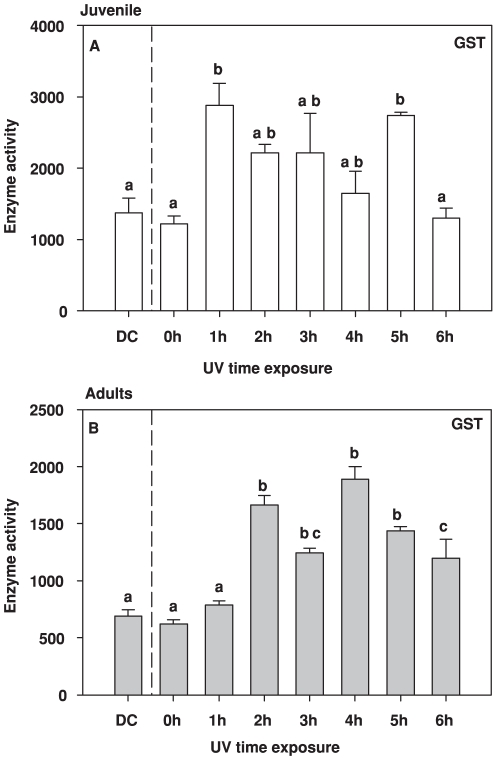
Glutathione *S*-transferase (GST) activity under UVR exposure for: (A) juveniles and (B) adults of copepod the *Eudiaptomus gracilis* under cumulative doses of UVR (0–6 h, DC = Dark Control). Letters inside the graphs indicate homogeneous groups (treatments with non-significant differences) of enzyme activity as shown by the *a posteriori* analysis. Activity is expressed in nmol product min^−1^ [mg proteins]^−1^.

The GST activity increased upon UVR exposure in both juvenile and adult copepod stages (Fig. 1) but the response was stronger in juveniles (two way ANOVA F_1,32_ = 56.43, *p*<0.001). An early response in GST was noticed in juveniles, with an elevated activity after only 1 h of exposure (two way ANOVA F _7,32_ = 9.51 *p*<0.001, Tukey *a posteriori* test DC versus 1 h *p* = 0.008, 0 h versus 1 h p = 0.002) (Fig. 1A). A longer time lag in the response was observed in adults, with no increase in the first hour, but a significant increase in activity after 2–5 h of UVR exposure compared the DC (Fig. 1B, two way ANOVA F _7,32_ = 9.51 *p*<0.001 Tukey test DC versus 0 h *p*>0.05, DC versus 1 h *p*>0.05, DC versus 2 h *p* = 0.033). The GST response after 2–5 h was stronger in juveniles than in adults (*t* test, *p* = 0.029) (Fig. 1).

**Figure 2:**
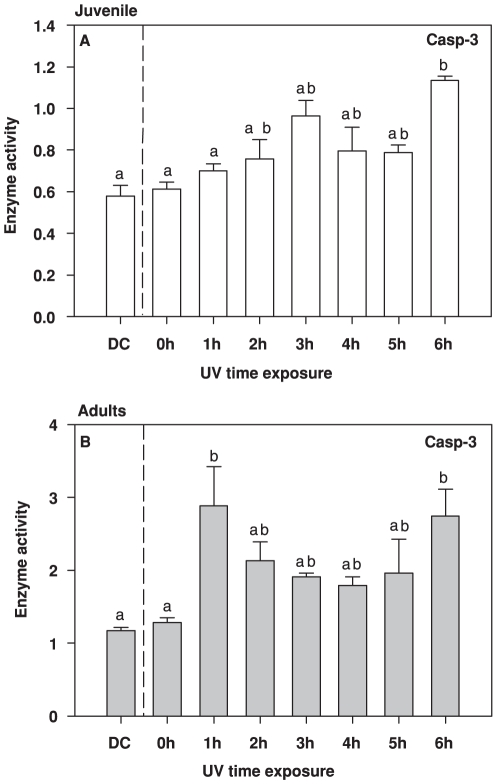
Caspase-3 (Casp-3) activity from juveniles (A) and adults (B) of the copepod *Eudiaptomus gracilis* under cumulative doses of UVR (0–6 h, DC = Dark Control). Letters inside the graphs indicate homogeneous groups (treatments with non-significant differences) of enzyme activity as shown by the *a posteriori* analysis. Activity is expressed in µmol product min^−1^ [mg proteins]^−1^.

The Casp-3 activity in response to UVR exposure was stronger in adults than in juveniles (two way ANOVA F_1,32_ = 116.95, *p*<0.001) (Fig. 2A and B). In juveniles, Casp-3 showed an increase during the exposure (UVR dose), reaching maximum levels at the end of the experiment. In adults, though, the activity of Casp-3 was not linear with UV dose, but there were significantly higher activities in the beginning (1 h) and the end of the exposure (6 h) than the DC and 0 h (Fig. 2B, two way ANOVA F_7,32_ = 4.84, *p*<0.001 Tukey test DC versus 1 h *p*<0.001, 0 h versus 1 h p<0.001, DC versus 6 h *p*<0.001 and 0 h versus 6 h p = 0.002).

**Figure 3:**
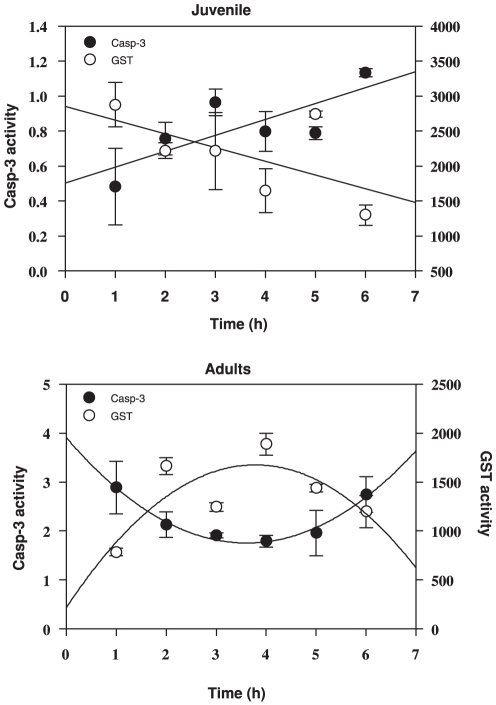
GST-Casp-3 relationship. GST activity was expressed in nmol product min^−1^ [mg proteins]^−1^ wereas Cap-3 activity in µmol product min^−1^ [mg proteins]^−1^.

The activities of these two enzymes, GST and Casp-3, showed opposite trends and different patterns. In juveniles, both enzymes showed linear responses, with a decrease in GST (Linear regression r^2^ = 0.231, F_1,16_ = 4.810, *p* = 0.0434) but an increase in Casp-3 (Linear regression r^2^ = 0.368, F_1,16_ = 9.320, *p* = 0.0076) (Fig. 3A). For adults, though, the observed pattern for both enzymes was a quadratic response—again contrasting—with a maximum of GST at 4 h (Quadratic regression r^2^ = 0.413, F_2,15_ = 5.283, *p* = 0.0183), and a simultaneous minimum activity for Casp-3 (Quadratic regression r^2^ = 0.527, F_2,15_ = 8.365, *p* = 0.0036) (Fig. 3B). Pearson's correlation confirmed a significant opposite trend between both enzyme activities in both stages (adults r^2^ = 0.329, *p* = 0.016 and juveniles r^2^ = 0.253, *p* = 0.047)

**Figure 4:**
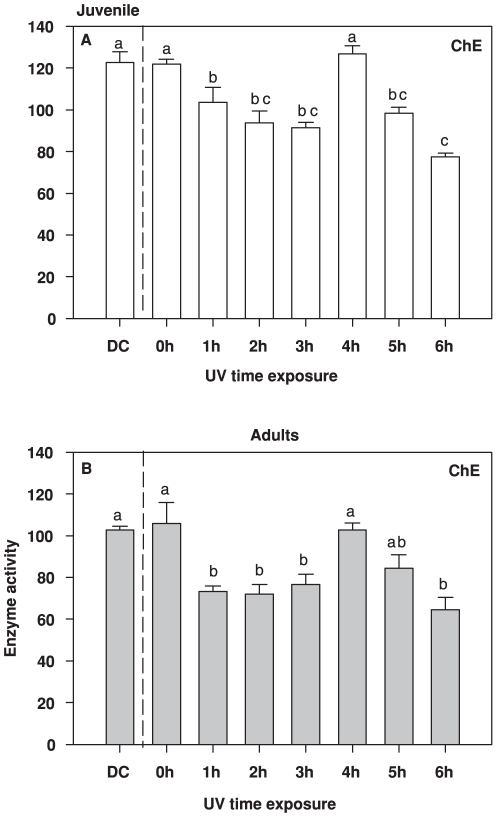
Cholinesterase (ChE) activity from juveniles (A) and adults (B) of the copepod *Eudiaptomus gracilis* under cumulative doses of UVR (0–6 h, DC = Dark Control). Letters inside the graphs indicate homogeneous groups (treatments with non-significant differences) of enzyme activity as shown by the *a posteriori* analysis. Activity is expressed in µmol product min^−1^ [mg proteins]^−1^.

We observed distinctive cumulative effects in ChE activity under increasing UVR doses (Fig. 4). The response pattern to UVR was similar between juveniles and adults and showed significant initial decrease at 1 h of exposure (about 20–30% reduction, two way ANOVA F_7,32_ = 14.67, *p*<0.001). There was a recovery to normal levels of activity after 4 h of exposure (Fig. 4A and B), and finally a new reduction in activity upon longer exposure. These enzymatic pattern was the same to both copepods' stages analyzed (Two way ANOVA F _7,32_ = 1.150, *p* = 0.358).

## Discussion

When intermittent environmental threats are part of an organism's existence, the defence that they mount may be either constitutive and permanent or inducible and temporary [29], [30], [37], [38], [39]. For these latter plastic responses, a lag phase of variable length is necessarily entailed [40], which means that during this phase the organism is relatively unprotected against the threat. Defences against UVR damage, such as pigments and other photoprotective compounds, are quite well known [6], [9], [24], [29], [41]. Yet studies focusing on short-term boosts of UVR exposure (minutes–hours), and the sublethal effects of such, are surprisingly rare, even though such fluctuations occur regularly in nature, e.g. due to daily changes in weather conditions and clouding. Here we show, for the first time, how short-term UVR exposure at similar to natural, sub-lethal levels simultaneously triggers responses in two different enzyme systems involved in key cellular processes: GST (antioxidant defence, Fig. 1), and Casp-3 (apoptosis, Fig. 2), and how this short exposure may affect physiological endpoints such as ChE (neurotransmitter control, Fig. 4).

Low levels of ROS (reactive oxygen species) are generated during normal oxidative processes in all aerobic organisms [42], and these low levels of free radicals are generally not harmful to the cell [43]. In moderate concentrations, ROS are necessary for a number of protective reactions, as it can act as molecular signals that trigger endogenous defence mechanisms and recently associated with increased resistance and longevity [44]. But with sudden increases in environmental stress, as can occur with UVR during a summer day, ROS levels rise dramatically resulting in potential oxidative damage [45]. A surplus of ROS causes alterations in crucial biomolecules: physical damage to DNA, carbonylation of proteins, and peroxidation of lipids [42]. Previous studies suggest that GST is a suitable signal of antioxidant responses in zooplankton [13], [14], [46]. Here we show that GST activity in copepods increase within two hours after UVR exposure, suggesting a rapid cellular response to stress.

Cellular response also implies changes in the apoptosis process. Casp-3 is one of the most studied caspases in crustaceans [47], and UVR has been indicated as one of the most important stimuli for apoptosis [15]. In our experiments, we observed an increase in Casp-3 enzymatic activity with UVR exposure, and it was particularly high in adults in the initial stages and after several hours of treatment. UVR causes severe DNA genomic damage by promoting formation of cyclobutane pyrimidine dimers and by increasing the concentrations of free radicals. If DNA repair mechanisms fail to correct the damage, the apoptosis cascade will be trigged to ensure that unwanted and potentially dangerous cells are efficiently removed, while cells that are transiently stressed by environmental conditions can recover and survive [18]. Thus, in multicellular organisms, apoptosis mechanisms could be considered part of the defence strategy against the detrimental impact of UVR.

It has been shown that UVR affects ChE activity in different copepod species [23] so we used this enzyme as a measure of cell damage. In the present study, a rapid decrease in ChE activity was found in *Eudiaptomus gracilis*, in spite of defence mechanisms. The enzyme activity decreased significantly over the course of the exposure, even though there was a recovery in activity after 4 h followed by another decrease. Such transient recovery has previoulsy been observed in copepods and other organisms [23], [48]. If the effect of the stressor persists (5–6 h of UVR exposure in this study) and the damage in the cell increases, ChE activity drops further. Adequate ChE activity levels are crucial for normal neuro-muscular function [22] and an impairment of ChE activity may therefore strongly affect physiological processes that are crucial for population fitness.

The time course curves for Casp-3 activity were the inverse of those for GST. The lag in the response of GST among adults likely constitutes a window of time when the cell is susceptible to UVR. The rapid increase in Casp-3 during the first hour suggests that the copepods are removing damaged cells. However, after two hours the level of GST activity increased with a concurrent decrease in Casp-3, indicating that the defence shift to antioxidant enzymatic quenching. Subsequently, when the cumulative doses of UVR eventually mounted up (4–6 h of exposure) and the GST among adults decreased, the apoptotic Casp-3 was again called upon, as seen by its increase in activity. A similar pattern was observed among juveniles, but since there was no detec lag in GST response, there was little need for removal of damaged cells in the initial stages of exposure. However, ChE activity decreases in this short time period (1 h) suggesting that there is threshold of damage that organisms cannot prevent. Even so, as UVR doses accumulated, the same pattern of decrease in GST and increase in Casp-3 was observed.

Here, we show that copepods have an enzymatic way to rapidly deal with short-term boosts in UVR exposure. Responses at molecular levels represent early warning signals and may provide useful information on how organisms respond to environmental stressors. Moreover, that several enzymes are involved suggests that neither of them is sufficient, but the combination of different enzyme systems would be necessary to reduce the stress experienced by the organism. Further experiments with mutant strains are needed to elucidate the interplay among these systems.

In conclusion, our study on short-term responses mimics natural conditions experienced by zooplankton during a day with fluctuating UVR threat. Because pigments and other photoprotective compounds require lag phases of days to weeks, copepods employ the adaptation of inducing their cellular enzyme systems that have much shorter lag phases. Hence, from an evolutionary perspective, the access to several different protective systems—behavioural (vertical migration), morphological (photoprotective pigmentation), as well as rapidly mobilized enzyme systems—may considerably improve protection from stresses such as UVR and thereby increase the animal's fitness.
